# Supplementary material for: The Impact of Cognitive Impairment on Treatment Toxicity, Treatment Completion, and Survival among Older Adults Receiving Chemotherapy: A Systematic Review
Source: Cancers (Basel). 2022 Mar 21;14(6):1582. doi: 10.3390/cancers14061582 (PMC8946153; doi:10.3390/cancers14061582)
Supplement: Supplementary file 1 [file cancers-14-01582-s001.zip › Supplementary S1_Search Strategies.pdf]

Ovid MEDLINE(R) and In-Process, In-Data-Review & Other Non-Indexed Citations and Daily 1946 to March 01, 2021

1. exp. Neoplasms/
2. (cancer* OR carcinoma*OR neoplas* OR tumor* OR tumour* OR malignan*).mp [mp=title, abstract, original title, name of substance word, subject heading word, floating sub-heading word, keyword heading word, organism supplementary concept word, protocol supplementary concept word, rare disease supplementary concept word, unique identifier, synonyms]
3. 1 or 2
4. exp. Aged/
5. (aging OR aged OR senior* OR geriatric* OR elder* OR old*).mp [mp=title, abstract, original title, name of substance word, subject heading word, floating sub-heading word, keyword heading word, organism supplementary concept word, protocol supplementary concept word, rare disease supplementary concept word, unique identifier, synonyms]
6. 4 or 5
7. exp. Neurocognitive Disorders/
8. (Alzheimer* OR dement*).mp [mp=title, abstract, original title, name of substance word, subject heading word, floating sub-heading word, keyword heading word, organism supplementary concept word, protocol supplementary concept word, rare disease supplementary concept word, unique identifier, synonyms]
9. cognit*.mp [mp=title, abstract, original title, name of substance word, subject heading word, floating sub-heading word, keyword heading word, organism supplementary concept word, protocol supplementary concept word, rare disease supplementary concept word, unique identifier, synonyms]
10. (dysfunct* OR impair* OR disorder* OR declin* OR deteriorat*).mp [mp=title, abstract, original title, name of substance word, subject heading word, floating sub-heading word, keyword heading word, organism supplementary concept word, protocol supplementary concept word, rare disease supplementary concept word, unique identifier, synonyms]
11. 9 and 10
12. 7 or 8 or 11
13. 3 and 6 and 12
14. Limit 13 to (English or Spanish)

APA PsycInfo (OVID) 1806 to March Week 1 2021

1. exp Older Adulthood/
2. exp Geriatric Patients/
3. exp Nursing Home Residents/
4. exp Geriatrics/
5. (old or older* or aged or aging or elder* or senior* or geriatric*).mp. [mp=title, abstract, heading word, table of contents, key concepts, original title, tests & measures, mesh]
6. 1 or 2 or 3 or 4 or 5
7. exp Neoplasms/

8. (cancer* or tumor* or tumour* or malignan* or carcinom* or neoplas*).mp. [mp=title, abstract, heading word, table of contents, key concepts, original title, tests & measures, mesh]
9. 7 or 8
10. 6 and 9
11. exp Cognitive Impairment/
12. exp Neurocognitive Disorders/
13. Alzheimer* or dement*.mp [mp=title, abstract, heading word, table of contents, key concepts, original title, tests & measures, mesh]
14. cognitiv*.mp. [mp=title, abstract, heading word, table of contents, key concepts, original title, tests & measures, mesh]
15. (dysfunct* or impair*).mp. [mp=title, abstract, heading word, table of contents, key concepts, original title, tests & measures, mesh]
16. 14 and 15
17. 11 or 12 or 13 or 16
18. 10 and 17
19. Limit 18 to (English or Spanish)

Embase 1974 to 2021 March 1 (OVID)

1. exp malignant neoplasm/

2. (cancer* or malignan* or neoplas* or tumor* or tumour* or carcinoma*).mp [mp=title, abstract, heading word, drug trade name, original title, device manufacturer, drug manufacturer, device trade name, keyword, floating subheading word, candidate term word]

3. 1 or 2

4. exp aged/

5. (aging or aged or senior* or old* or geriatric* or elder*).mp [mp=title, abstract, heading word, drug trade name, original title, device manufacturer, drug manufacturer, device trade name, keyword, floating subheading word, candidate term word]

6. 4 or 5

7. 3 and 6

8. exp cognitive defect/

9. exp “disorders of higher cerebral function”/

10. (dement* or Alzheimer*).mp [mp=title, abstract, heading word, drug trade name, original title, device manufacturer, drug manufacturer, device trade name, keyword, floating subheading word, candidate term word]

11. cognitiv*.mp [mp=title, abstract, heading word, drug trade name, original title, device manufacturer, drug manufacturer, device trade name, keyword, floating subheading word, candidate term word]

12. dysfunct* or disorder* or impair*).mp [mp=title, abstract, heading word, drug trade name, original title, device manufacturer, drug manufacturer, device trade name, keyword, floating subheading word, candidate term word]

13. 11 and 12

14. 8 or 9 or 10 or 13

15. 7 and 14

16. limit 15 to (English or Spanish)

CINAHL Plus with Full Text (Ebscohost)

S1 (MH “Aged+”)

S2 aged OR elder* OR senior* OR geriatric* OR old OR older

S3 S1 OR S2

S4 (MH “Neoplasms+”)

S5 (MH “Cancer Patients”)

S6 cancer* OR carcinoma* OR tumour* OR tumor* OR malignan*

S7 S4 OR S5 OR S6

S8 (MH “Delirium, Dementia, Amnestic, Cognitive Disorders+”)

S9 alzheimer* OR dement*

S10 cognitiv*

S11 impair* OR dysfunct* OR disorder*

S12 S10 and S11

S13 S8 OR S9 OR S12

S14 S3 AND S7 AND S13

S15 Limit S14 to (English OR Spanish)
