# Supplementary material for: The Impact of Cognitive Impairment on Treatment Toxicity, Treatment Completion, and Survival among Older Adults Receiving Chemotherapy: A Systematic Review
Source: Cancers (Basel). 2022 Mar 21;14(6):1582. doi: 10.3390/cancers14061582 (PMC8946153; doi:10.3390/cancers14061582)
Supplement: Supplementary file 1 [file cancers-14-01582-s001.zip › Supplementary S2_Risk of Bias Assessment.pdf]

Supplementary S2. Risk of Bias Assessment*

Author	Study participation	Study attrition	Prognostic factor measurement	Outcome measurement	Study confounding	Statistical analyzing and reporting
**Abe (2011)** [24]	Moderate	Low	Moderate	Low	Moderate	Low
**Aaldriks (2011)** [25]	Low	Low	Low	Low	Low	Low
**Aaldriks (2013a)** [26]	Low	Low	Low	Low	Low	Low
**Aaldriks (2013b)** [27]	Low	Low	Low	Low	Low	Low
**Aaldriks (2016)** [28]	Low	Not applicable	Low	Low	Low	Low
**Aparicio (2013)** [29]	Low	Not applicable	Moderate	Low	Low	Low
**Biesma (2011)** [30]	Moderate	Low	Low	Moderate	Low	Low
**Dubruille (2015)** [31]	Low	Low	Low	Low	Low	Low
**Extermann (2012)** [32]	Low	Moderate	Low	Low	Low	Low
**Falandry (2013a)** [33]	Low	Low	Low	Moderate	Low	Moderate
**Falandry (2013b)** [34]	Low	Low	Low	Moderate	Low	Moderate
**Hamaker (2014)** [35]	Low	Moderate	Low	Low	Low	Low
**Hshieh (2018)** [3]	Moderate	Low	Low	Low	Low	Low
**Jayani (2019)** [12]	Low	Low	Low	Low	Low	Low
**Klepin (2013)** [36]	Low	Low	Low	Low	Low	Low
**Laurent (2014)** [37]	Low	Moderate	Moderate	Low	Not applicable	Low
**Lee (2020)** [44]	Low	Low	Moderate	Low	Not applicable	Low
**Molga (2019)** [38]	Low	Low	Low	Low	Low	Low
**Robb (2009)** [49]	Low	Low	Low	Low	Low	Low
**Shin (2012)** [40]	Low	Moderate	Low	Low	Low	Low
**Soubeyran (2012)** [41]	Low	Moderate	Low	Low	Low	Low
**Thibaud (2021)** [42]	Low	Low	Moderate	Low	Moderate	Low
**Wildes (2013)** [43]	Low	Low	Low	Low	Low	Low

*Based on the Quality Assessment in Prognostic Studies (QUIPS) tool
