# Supplementary material for: The Impact of Cognitive Impairment on Treatment Toxicity, Treatment Completion, and Survival among Older Adults Receiving Chemotherapy: A Systematic Review
Source: Cancers (Basel). 2022 Mar 21;14(6):1582. doi: 10.3390/cancers14061582 (PMC8946153; doi:10.3390/cancers14061582)
Supplement: Supplementary file 1 [file cancers-14-01582-s001.zip › Supplementary S3_Results from Individual Studies.pdf]

**Supplementary S3. Results from Individual Studies**

| Author/year | Proportion with and/or Degree of cognitive impairment | Endpoint outcome(s) examined | Duration of follow-up | Baseline dose reduction (Y/N) | Found significant (Y/N) | Details | Multivariate model performed for CI (Y/N) | Adjusted for in MV |
|---|---|---|---|---|---|---|---|---|
| **Survival/Mortality** | | | | | | | | |
| Abe[24] | 22.6%) had CI (7/31) CI<br><br>N=4 Mild (MMSE: 20-23)<br>N=3 Moderate (MMSE: 14-19) | 1-year Overall Survival | 1 year | NR | N | 1-year OS of patients with CI was 0%.<br>In univariate analysis model, CI[a] was not associated with 1-year OS (p=0.1384) (no other estimates provided) | N/A | N/A |
| Aldricks[25] (2011) | 10% (21/202) had CI (MMSE <=24): | Mortality | Median 9 months (1-33) | NR | N | Model 1: HR 0.99 (0.48-2.07)<br><br>Model 2: HR 0.92 (0.44-1.93) | N/A | N/A |
| Aaldriks (2013a)[26] | 13% (19/143) suspect for cognitive decline (IQ-CODE >3.31 pts) | Mortality | Median 15 months (0.5-62) | 16% patients received reduced dose (reason not known. It is not reported how many % of which were patients with CI) | N | Adjuvant group: HR 3.08 (0.76-20.93)<br><br>Palliative group: HR 1.51 (0.59-3.85) | N/A | N/A |

| Author/year | Proportion with and/or Degree of cognitive impairment | Endpoint outcome(s) examined | Duration of follow-up | Baseline dose reduction (Y/N) | Found significant (Y/N) | Details | Multivariate model performed for CI (Y/N) | Adjusted for in MV |
|---|---|---|---|---|---|---|---|---|
| | 8% (11/143) had CI (MMSE <= 24 pts): | | | | | | | |
| Aaldriks (2013b)[27] | 9% (5/55) had CI (MMSE <= 24 pts):<br><br>18% (10/55) had CI (IQCODE >= 3.3) | Mortality | Median 11 months (0-57) | NR | N | MMSE: HR 1.68 (0.49-5.78)<br><br>IQCODE: HR 1.07 (0.49-2.37) | N/A | N/A |
| Aaldriks (2016)[28] | 12.9% (62/494) suspect for cognitive decline (IQ-CODE >3.31 pts)<br><br>9% (44/494) had CI (MMSE <= 24 pts) | Mortality | Time between first geriatric assessment (window from May 2004-Feb 2010) and January 1, 2013, or the date of death | NR | N | MMSE: 1.36 (0.97-1.91)<br><br>IQCODE: 1.12 (0.83-1.50) | N/A | N/A |

| Author/year | Proportion with and/or Degree of cognitive impairment | Endpoint outcome(s) examined | Duration of follow-up | Baseline dose reduction (Y/N) | Found significant (Y/N) | Details | Multivariate model performed for CI (Y/N) | Adjusted for in MV |
|---|---|---|---|---|---|---|---|---|
| | | | | | | | | |
| Biesma[30] (2011) | NR | Survival | Median 21.0 months | NR | N | HR 0.95 (0.83-1.09) | N/A | N/A |
| Dubruille[31] (2015) | 31% (28/90) had CI (MMSE <27)<br><br>51% (46/90) had CI (MoCA <26)<br><br>Patients with dementia | One-year survival | 1 year | Initial treatment choice: 66% received full dose, 34% received reduced ode. However, unclear how many CI patients received which dose. | Y | Cognitive status (HR = 3.260, 95% CI: 1.043-10.194; p = 0.042) were predictive of OS<br><br>1-year OS was 63% for patients with CI versus 88% for those without. | Y | Age, gender, diagnosis, disease status, ECOG, initial treatment choice, tolerance to treatment |

| Author/year | Proportion with and/or Degree of cognitive impairment | Endpoint outcome(s) examined | Duration of follow-up | Baseline dose reduction (Y/N) | Found significant (Y/N) | Details | Multivariate model performed for CI (Y/N) | Adjusted for in MV |
|---|---|---|---|---|---|---|---|---|
| | were excluded | | | | | | | |
| Falandry[33] (2013a) | 29% (32/111) had CI (MMS <25) | Overall survival | Median 16.4 months (0.2-49.6 months) | Patients received carbo dosed at AUC 5mg/ml/min for 30 min q3 week up to 6 cycles | N | MMSE score >24 was associated with increased survival: HR 1.08, p=0.79 (no confidence interval provided) | N/A | N/A |
| Falandry[34] (2013b) | Five words recall: mean 18.5 (SD 3.4) (no other details provided) | Progression free survival (PFS) and overall survival | 24 months | Did not mention<br><br>Only said 6 courses of “adapted dose of pegylated liposomo doxorubisin(40mg/m Q28 days)” | N | NR | N/A | N/A |
| Hamaker[35] | 7% MMSE<=23 | Survival | 24 months | NR | Y<br><br>N | Univariate model: HR 3.74 (1.43-9.73)<br>Multivariate model: HR 1.88 (confidence interval not provided) | Y | Age, performance status, chemotherapy type |

| Author/year | Proportion with and/or Degree of cognitive impairment | Endpoint outcome(s) examined | Duration of follow-up | Baseline dose reduction (Y/N) | Found significant (Y/N) | Details | Multivariate model performed for CI (Y/N) | Adjusted for in MV |
|---|---|---|---|---|---|---|---|---|
| Hshieh[3] (2018) | 35.3% (127/360) had probable executive dysfunction (CIB score <5);<br><br>17.2% (62/360) had probable working memory impairment (<= 2 words in 5-word delayed recall | Survival | 1 year | 65.6% percent of sample underwent intensive treatment: among this group decreased survival was seen for probable impairment For both tests. | Y | Patients with impaired working memory had worse median survival (10.9 [SD 12.9] vs 12.2 [SD 14.7] months; log rank p< .001), including when stratified by indolent cancer (log rank *p* = .01) and aggressive cancer (*p* < .001), and in multivariate analysis when adjusted for age, comorbidities, and disease aggressiveness (adds ratio, 0.26; 95% CI, 0.13-0.50). Impaired working memory was also associated with | Y | Age, comorbidity, cancer aggressiveness |

| Author/year | Proportion with and/or Degree of cognitive impairment | Endpoint outcome(s) examined | Duration of follow-up | Baseline dose reduction (Y/N) | Found significant (Y/N) | Details | Multivariate model performed for CI (Y/N) | Adjusted for in MV |
|---|---|---|---|---|---|---|---|---|
| | | | | | | worse survival for those undergoing intensive treatment (log-rank *p* < .001) Executive dysfunction was associated with worse survival only among patients who underwent 'intensive treatment' (log-rank *p* = .03) | | |
| Klepin[36] (2013) | 28.8% had CI (3MS** < 77) | Overall survival | 30 days | No mention of dose reduction | Y | Impaired cognition was associated with worse overall survival Unadjusted HR 2.4 (95% CI: 1.3-4.4) Adjusted^^ HR 2.5 (95% CI: 1.2-5.5) | Y | Age, gender, ECOG, cytogenic risk group, MDS, hemoglobin |

| Author/year | Proportion with and/or Degree of cognitive impairment | Endpoint outcome(s) examined | Duration of follow-up | Baseline dose reduction (Y/N) | Found significant (Y/N) | Details | Multivariate model performed for CI (Y/N) | Adjusted for in MV |
|---|---|---|---|---|---|---|---|---|
| | | | | | | median OS for patients with CI was 5.2 months compared to 15.6 months for those without impairment | | |
| Lee[44] (2020) | 28.3% had dementia (no details on assessment tool used) | Overall survival | Median 15.4 months (0.3-107.6) | Unclear | N | No significant difference was found between survivor group and non-survivor group in terms of cognitive status (p=0.168). Confidence interval not provided | N/A | N/A |
| Molga[38] (2019) | 11% had CI (MMSE < 24) | Overall survival | Varied | N | N | HR 1.08 (0.38-3.02)<br><br>Analysis of OS of those who could not complete 6 cycles included 8 patients – the 4 | N/A | N/A |

| Author/year | Proportion with and/or Degree of cognitive impairment | Endpoint outcome(s) examined | Duration of follow-up | Baseline dose reduction (Y/N) | Found significant (Y/N) | Details | Multivariate model performed for CI (Y/N) | Adjusted for in MV |
|---|---|---|---|---|---|---|---|---|
| | | | | | | with CI and 4 others without CI). The 4 patients with CI stopped due to comorbidities or intercurrent illness; whereas patients without CI stopped due to lack of response/disease progression | | |
| Robb[39] (2009) | Case control study: case: n=86 Control: n=172<br><br>Among the cases, a cutoff of <=20 was used to delineate mild vs. moderate-to-severe | Survival | N/A (case-control) | NR | Y | Non-CI group had greater survival (Mdn = 72.6 months) than CI group (Mdn = 23.0 months); $p<.001$<br><br>Cognitive status was an independent predictor of survival (p=.045) (other estimates not available) | Y | Baseline ECOG, ADLs/IADLs, CIRS-G, Charlson scores, GDS score, age, and gender |

| Author/year | Proportion with and/or Degree of cognitive impairment | Endpoint outcome(s) examined | Duration of follow-up | Baseline dose reduction (Y/N) | Found significant (Y/N) | Details | Multivariate model performed for CI (Y/N) | Adjusted for in MV |
|---|---|---|---|---|---|---|---|---|
| Soubeyran[41] (2012) | 19% had CI (MMSE <= 23) | Mortality (early death risk [defined as within 6 months]) | 6 months | 14.9% received standard reduced treatment<br><br>40.2% received adapted treatment Unclear how many of whom among these groups had CI | Y<br><br>N | Significant in univariate model (P = .012) (actual magnitude effects not provided)<br><br>Not significant in multivariate model adjusting for treatment site (details NR) | Y | Treatment site |
| Thibaud[42] (2021) | 31% had CI (MMSE <27) | Survival | 12 months | 65% received standard dose: 35% received reduced dose. Unclear whether or how many CI patients had reduced dose | Y | Cognitive impairment (MMSE < 27) predicted one-year survival (HR 1.82 [95% CI 1.13-2.91], *p* = 0.01)<br><br>(actual magnitude of effects not provided) | Y | "*impact of disease itself and other vulnerabilities*" |
| Wildes[43] (2013) | 4.8% (3/63) | Mortality | Median 20 months (0.01-47 months) | n=1 received dose reduction due to renal impairment | N | HR 0.83 (0.11-6.23) | N/A | N/A |

| Author/year | Proportion with and/or Degree of cognitive impairment | Endpoint outcome(s) examined | Duration of follow-up | Baseline dose reduction (Y/N) | Found significant (Y/N) | Details | Multivariate model performed for CI (Y/N) | Adjusted for in MV |
|---|---|---|---|---|---|---|---|---|
| | Short Blessed score >9 | | | | | | | |
| **Chemotherapy completion** | | | | | | | | |
| Abe[24] (2011) | 22.6% had CI:<br><br>N=4 Mild (MMSE: 20-23)<br>N=3 Moderate (MMSE: 14-19) | Complete remission (CR)<br><br>Unplanned endpoint: discontinuation of consolidation chemotherapy | 3 year | All 7 alzeihmers pateints received (donezepezil hydrochloride, drug therapy for BPSD, and support by family during chemotherapy.<br><br>No mention of dose reduction | Unclear | All 7 patients with CI were able to receive chemotherapy through administration of donepezil hydrochloride, and family support during chemotherapy administration. CR was achieved in the 3 moderate cases CI.<br>However, consolidation chemotherapy was discontinued in all 7 patients with CI due to | N/A | N/A |

| Author/year | Proportion with and/or Degree of cognitive impairment | Endpoint outcome(s) examined | Duration of follow-up | Baseline dose reduction (Y/N) | Found significant (Y/N) | Details | Multivariate model performed for CI (Y/N) | Adjusted for in MV |
|---|---|---|---|---|---|---|---|---|
| | | | | | | exacerbation of BPSD | | |
| Aaldriks[28] (2016) | 12.9% had suspect for cognitive decline (IQ-CODE >3.31 pts)<br>9% had CI (MMSE <= 24 pts): 9% | Chemotherapy completion (>= 4 cycles) | Time between first geriatric assessment (window from May 2004-Feb 2010) and January 1, 2013, or the date of death | It was only reported that patients stated chemotherapy.<br><br>No mention of dose reduction | N | Univariable analysis:<br><br>IQCODE:<br>OR 0.96 (0.53-1.75)<br><br>MMSE:<br>OR 1.65 (0.87-3.13) | N/A | N/A |
| Aldricks[25] (2011) | 11% had CI (MMSE <=24)<br><br>15% had CI (IQCODE >3.31) | Chemotherapy completion | Unclear | NR | Y | Compared to those who received >=4 cycles of chemotherapy, MMSE scores were significant lower for patients who received <4 cycles (p=0.04) | N/A | N/A |

| Author/year | Proportion with and/or Degree of cognitive impairment | Endpoint outcome(s) examined | Duration of follow-up | Baseline dose reduction (Y/N) | Found significant (Y/N) | Details | Multivariate model performed for CI (Y/N) | Adjusted for in MV |
|---|---|---|---|---|---|---|---|---|
| | | | | | | (other estimates not available)<br><br>Among those with CI (IQCODE >3.31), 20% underwent <4 cycles whereas 13% underwent >=4 cycles (p=0.2).<br><br>Among those with CI (MMSE <= 24), 11% underwent <4 cycles whereas 3% underwent >=4 cycles (p=0.04). | | |
| Laurent[37] (2014) | 12.3% had CI (MMSE<24) | Chemo discontinuation ?<br>(Chemo feasibility??) | Median 13.4 months (5.3-24.6) | NR | Y | Higher MMSE (median 28 (27-30]) significant in univariate analysis (p = .05) | Y | Functional status, mobility limitation f |

| Author/year | Proportion with and/or Degree of cognitive impairment | Endpoint outcome(s) examined | Duration of follow-up | Baseline dose reduction (Y/N) | Found significant (Y/N) | Details | Multivariate model performed for CI (Y/N) | Adjusted for in MV |
|---|---|---|---|---|---|---|---|---|
| | | | | | N | (actual absolute magnitude of effects not provided)<br>Not significant in multivariate model (details on estimates not provided) | | |
| Molga[38] (2019) | 11% had CI (MMSE <24) | Chemotherapy completion | 50 months | It was mentioned there was no change in planned treatment due to geriatric deficits. | Y | Patients with CI completed significantly less azacitidine cycles compared to those without CI (3.5 ± 2.1 vs. 10.9 ± 7.9; p=0.34)<br><br>A higher number of patients with CI (3/4; 75%) could not completed 6 cycles of azacitidine compared to patients without | N/A | N/A |

| Author/year | Proportion with and/or Degree of cognitive impairment | Endpoint outcome(s) examined | Duration of follow-up | Baseline dose reduction (Y/N) | Found significant (Y/N) | Details | Multivariate model performed for CI (Y/N) | Adjusted for in MV |
|---|---|---|---|---|---|---|---|---|
| | | | | | | CI (5/21; 24%) (p = .05). | | |
| Wildes[43] (2013) | 4.8% had severe CI (Short Blessed score >9) | Chemo completion | Median 20 months (0.01-47 months) | n=1 received dose reduction due to renal impairment | N | OR 0.71 (0.06-8.46) | N/A | N/A |
| **Chemotherapy toxicity** | | | | | | | | |
| Aparicio[29] (2013) | 31% had CI (MMSE <= 27) | Treatment toxicity (at least one grade 3-4 toxicity) | The three outcomes were analyzed during the first 4 months of starting treatment | Randomized to FU-based chemotherapy alone or in combination with IRI. In the IRI arm, first 2 cycles were 150mg/m of IRI, and increased in the absence of toxicity in subsequent cycle. | Y | MMSE (<=27) was significant predictive factor in multivariate analysis for grade 3-4 toxicity (OR 3.84; 95% CI: 1.24-11.84)<br><br>In patients with MMSE <=27/30, 89% 9n=17) in the IRI arm had grade 3-4 toxicity versus 50% (n=9) in the FU arm. In patients with an MMSE >27/30, | Y | NR |

| Author/year | Proportion with and/or Degree of cognitive impairment | Endpoint outcome(s) examined | Duration of follow-up | Baseline dose reduction (Y/N) | Found significant (Y/N) | Details | Multivariate model performed for CI (Y/N) | Adjusted for in MV |
|---|---|---|---|---|---|---|---|---|
| | | | | | | 58% (n=15) in the IRI arm had grade 3-4 toxicity versus 30% (n=8) in the FU arm | | |
| Extermann[32] (2012) | MMS score mean 28 (15-30)<br><br>Patients with dementia were excluded | Chemotherapy toxicity | Maximum 1 month after last cycle. IF chemotherapy was continued beyond 6 months, then follow-up was ended at 6 months | Gemcitabine 1.25g/m on day 1, a, 15 Q4 weeks<br><br>Chemo determined by oncologists by baseline. No mention whether patient received standard or dose-reduced treatment | Y<br><br><br><br>N | Association present for Grade 3/4 non hematologic toxicity: OR = 0.77 (0.63-0.93), p = .008<br><br>(Did not provided actual absolute magnitude of effects)<br><br>Association not present for grade 4 hematologic toxicity<br>OR 0.97 (0.87-1.07) | Y | Chemotherapy regimen |

| Author/year | Proportion with and/or Degree of cognitive impairment | Endpoint outcome(s) examined | Duration of follow-up | Baseline dose reduction (Y/N) | Found significant (Y/N) | Details | Multivariate model performed for CI (Y/N) | Adjusted for in MV |
|---|---|---|---|---|---|---|---|---|
| | | | | | | | | |
| Falandry[34] (2013b) | Five words recall<br><br>Mean 18.5 (SD 3.4) | Chemo toxicity<br><br>Chemo tolerance and efficacy outcomes<br><br>Tumor response | 24 months | NR | N | N/A | N/A | N/A |
| Hamaker[35] (2014) | 7% had CI (MMSE<=23) | Chemotherapy toxicity | Median 32 months | No mention of any baseline dose reduction<br><br>Randomized to doxorubicin 45mg/m q 4 weeks up to 6 weeks, or capecitabine 2000mg/m orally on days 1-14, every 3 weeks for up to 8 cycles | N | OR 1.14 (0.09-10.17) | N/A | N/A |
| Jayani[3] (2019) | 36% (250/703) had potential CI (BOMC score 5-10) | Chemotherapy toxicity | ? | 76% received standard dose (i.e. 24% had dose reduction. However, it is not known how | Y | Potential CI (BOMC score 5-10) was associated with increased risk of severe toxicity | Y | CARG Toxicity Risk Group (low, medium, high) |

| Author/year | Proportion with and/or Degree of cognitive impairment | Endpoint outcome(s) examined | Duration of follow-up | Baseline dose reduction (Y/N) | Found significant (Y/N) | Details | Multivariate model performed for CI (Y/N) | Adjusted for in MV |
|---|---|---|---|---|---|---|---|---|
| | Patients with dementia were excluded from analysis | | | many of whom had CI) | N | (OR 1.54, p<= 0.01). This was no longer significant after adjusting for CARG score; OR 1.98 (0.76-5.17) However, among patients with lower education level (n=258; 36.7%), potential CI remained associated with severe chemotherapy toxicity despite adjusting for CARG score (OR 1.08; p = 0.03)<br><br>The incidence of severe chemotherapy toxicity was 51.0% in patients without CI whereas the | | |

| Author/year | Proportion with and/or Degree of cognitive impairment | Endpoint outcome(s) examined | Duration of follow-up | Baseline dose reduction (Y/N) | Found significant (Y/N) | Details | Multivariate model performed for CI (Y/N) | Adjusted for in MV |
|---|---|---|---|---|---|---|---|---|
| | | | | | | incidence was 61.6% in patients with potential CI. | | |
| Shin[40] (2012) | Mild cognitive decline (MMSC-KC <=24): 51.6%<br><br>CI (MMSC-KC <16 signifies cognitive impairment): 4.7%<br><br>Patients with 'significant' CI were excluded | Chemo toxicity | Mean 8.89 weeks | NR | N | Mild CI: OR 0.68 (0.18-2.63)<br><br>CI: OR 1.53 (1.12-19.88) | N/A | N/A |
| Wildes[43] (2013) | 4.8% had severe CI( | Chemo toxicity (non-hematologic) | Median 20 months (0.01-47 months) | n=1 received dose reduction due to renal impairment | Not estimable due to 0 count of | N/A | N/A | N/A |

| Author/year | Proportion with and/or Degree of cognitive impairment | Endpoint outcome(s) examined | Duration of follow-up | Baseline dose reduction (Y/N) | Found significant (Y/N) | Details | Multivariate model performed for CI (Y/N) | Adjusted for in MV |
|---|---|---|---|---|---|---|---|---|
| | Short Blessed score >9) | | | | toxicity in the cell | | | |

[b]Abe et al. referred to mild and moderate CI as 'dementia' in this study.

**modified mini mental status exam

^^ adjusting for age, gender, hemoglobin, ECOG score, prior myelodysplastic syndrome, and cytogenic group

BOMC = Blessed Orientation-Memory-Concentration test

BPSD = Behavioural and psychological symptoms of dementia

CARG = Cancer and Aging Research Group
